# Potential Traumatic Events through the Life Cycle in an Immigrant Population

**DOI:** 10.3390/geriatrics6020039

**Published:** 2021-04-08

**Authors:** Gabriella C. Dong, Mengting Li

**Affiliations:** 1 Princeton High School, Princeton, NJ 08540, USA; 2 Institute for Health, Health Care Policy and Aging Research, Rutgers, The State University of New Jersey, New Brunswick, NJ 08901, USA; mengting.li@rutgers.edu; 3 School of Nursing, Rutgers, The State University of New Jersey, Newark, NJ 07102, USA

**Keywords:** life events, natural disasters, personal traumatic events, historical events

## Abstract

Existing studies on traumatic events focused on children, while it has been understudied in older adults. This study aims to examine prevalence, frequency, and severity of life events in older Chinese Americans. The data were drawn from the Population Study of Chinese Elderly (PINE) in 2017–2019. Twenty life events were evaluated, including natural disasters, personal traumatic events, and historical events. Among 3125 participants, the mean age was 75.33 (standard deviation (SD) = 8.22) with 61.06% female. Cultural Revolution (73.27%) has the highest prevalence. A total of 1819 (58.39%) participants reported typhoon and experienced multiple times. Falsely accused of crime was reported as the most severe event. Women were more likely to report family-related life events. Those with higher education were more likely to report personal traumatic events. This study is among the first to profile life events in older Chinese Americans. Age cohorts, gender roles, and socioeconomic status shape individuals’ exposure to life events. This study could help identify which vulnerable groups have high risks of exposure to traumatic events.

## 1. Introduction

Cumulative traumatic events could have substantial effects on physical and mental health. Prior empirical studies have found that exposure to these adversities could substantially increase the risk for depression, post-traumatic stress disorder (PTSD), anxiety, and cognitive impairment [1,2,3,4]. The experience of potential traumatic life events has also been identified to influence immense effects on activities of daily living (ADL), instrumental activities of daily living (IADL), and medical comorbidities [5]. Understanding the profile of life events among aging populations could help identify vulnerable subpopulations and develop tailored prevention and interventions to promote healthy aging.

Many prior studies have predominantly examined experience of traumatic events in childhood [4] and youth [6]. However, there are a limited number of studies that have examined lifetime exposure to traumatic events among older adults [5,7,8,9,10,11,12,13,14,15]. A few studies examined lifetime traumatic events among older Americans but lumped different ethnic groups together [5,7,8,9]. Different ethnic groups have different socioeconomic status in the United States and hold various cultural norms, which may influence their risks of experiencing traumatic events over the life course. Chinese immigrants constitute the largest group of Asian immigrants and their lifetime exposure to traumatic events include experiences in China that occurred before immigration and experiences in the United States after immigration. One study examined lifetime traumatic events among Chinese older adults in Singapore [16]. Life events experienced by Chinese older adults in Western countries might be different than those living in East Asia.

While many investigators examined the negative impacts of cumulative traumatic events on health, factors associated with traumatic events have not received enough attention. A few studies have shown that age, gender, and social economic status may influence the exposures to traumatic events [5,17]. Increased understanding of factors associated with exposure to traumatic events could inform preventive interventions and anticipate service needs [18].

One of the oldest, largest, and fastest growing Asian populations in the United States is Chinese American [19]. The life of Chinese immigrants in the United States has been affected by past anti-Chinese sentiment, U.S. immigration policies, and recent anti-Chinese sentiment during Covid-19. Due to language and cultural barriers, U.S. Chinese older adults are likely to live in Chinatown and have limited access to proper health care services and social services and have limited social engagement [20,21,22]. However, little is known about their lifetime exposures to traumatic events.

To fill this research gap, this study aims to provide a descriptive epidemiology of experience of lifetime traumatic events in older Chinese Americans. Specifically, this study examined (1) the prevalence, frequency, and severity of life events across the lifespan; and (2) the demographic factors related to lifetime traumatic events among older Chinese Americans.

## 2. Materials and Methods

### 2.1. Sample

The Population Study of Chinese Elderly (PINE) is a community-engaged, population-based longitudinal study of older Chinese Americans aged 60 and above in the Greater Chicago area [23]. The baseline PINE study was from 2011 to 2013 and utilized culturally appropriate community recruitment strategies guided by a community-based participatory research (CBPR) approach to ensure adequate community participation [24,25,26]. According to the U.S. census, approximately 1.6% of the households in Chicago contain a Chinese individual [24]. Given the high levels of concentration of Chinese Americans in Chinatown, targeted CBPR approach was implemented by first engaging community centers as the main recruitment site throughout the Greater Chicago area. Through sharing outreach channels and shared experiences with community centers, the research team was able to identify, outreach, and extend recruitment to eligible older adults in a vast area of the Greater Chicago area. Face-to-face home interviews were conducted by trained bicultural and bilingual interviewers. All participants were consented and interviewed in their preferred language, either Chinese (Mandarin or Cantonese) or English. In a comparison between the PINE study, the 2010 US Census data, and the 2012 Random Block Census study, we found that the PINE sample is representative of the Chinese older adults in the Greater Chicago area with no significant differences in sociodemographic and socioeconomic characteristics among these studies [27]. Follow-ups with participants occurred every two years. Successive cohorts were enrolled to account for attrition. In PINE T4 data collection, research questions on life events were inserted to the survey. This study used PINE T4 data collected in 2017–2019 with a sample size of 3125. The study was approved by the institutional review board at Rush University Medical Center in Chicago, Illinois (approval number: 10090203). Written informed consent was obtained from all participants.

### 2.2. Measurements

Potential traumatic events were evaluated by natural disasters, personal traumatic events, and historical events. Natural disasters included typhoons, earthquakes, and tornados. Personal traumatic events consisted of death of a loved one, being robbed, physical assault, residential fire, divorce, cancer, false accusation of a crime, homelessness, sexual assault, imprisonment, abortion, and miscarriage. The life events evaluated in our study are adopted from the traumatic life events questionnaire [28] and Harvard Trauma Questionnaire [29]. Two broad categories captured in the two scales were natural disasters and personal traumatic events. We further added five historical events, which are likely to be experienced by this cohort of Chinese older adults.

In terms of lifetime natural disasters and personal traumatic events, the prevalence, frequency, and severity for each event were evaluated. The prevalence was measured by asking participants “Have you ever personally experienced any of the following events?”. Frequency was assessed by asking participants “How many times did it happen in your life?”. Severity of the traumatic life events was assessed through asking “How serious do you think this event was/did it have a significant impact on you?”. The answer was rated from “not serious” = 1 to “very serious” = 3. Historical life events encompassed the Cultural Revolution, the Great Leap Forward, famine, Japanese invasion of China, and the Vietnam War. The prevalence and severity of each historical event were assessed.

Sociodemographic variables included age, gender (self-reported), income, and education (years of schooling). Annual personal income has been divided into ten categories (from 1 = $0–$4999 to 10 = $75,000 and over).

### 2.3. Data Analysis

The prevalence, frequency, and severity of personal life events, historical events, and natural disasters were presented with descriptive statistics. We used N (%) to describe the prevalence, frequency, and severity of the life events. Spearman correlation coefficients for age, gender, education, income, natural disasters, personal life events, and historical events were generated to identify correlates of life events. Statistical analyses were conducted using SAS, Version 9.2 (SAS Institute Inc., Cary, NC, USA).

## 3. Results

### 3.1. Sample Characteristics

The PINE study interviewed 3125 Chinese participants in the Greater Chicago area with a mean age of 75.33 (standard deviation (SD) = 8.22) and 61.06% was female. The average education was 9.23 years (SD = 4.89). Most (81.06%) participants had an average annual income below $10,000.

### 3.2. Prevalence, Frequency, and Severity of Life Events

Table 1 shows the prevalence of natural disasters, personal traumatic events, and historical life events in the sample. After examining the lifetime prevalence of natural disasters, we found typhoon (64.45%) had the highest prevalence, followed by earthquake (39.79%) and tornado (7.25%). In terms of personal traumatic events, death of a loved one (69.77%) was the most prevalent, followed by robbery (12.54%), physical assault (5.36%), residential fire (5.29%), divorce (5.17%), cancer (5.10%), falsely accused (2.15%), homeless (1.57%), sexual assault (0.99%), and imprisonment (0.74%). In addition, 18.92% of women experienced abortion and 11.26% of women experienced miscarriage. With respect to historical events, over half of participants experienced the Cultural Revolution (73.26%), the Great Leap Forward (62.73%), and famine (60.03%). A smaller proportion experienced the Japanese invasion of China (27.15%) and the Vietnam War (4.78%).

Table 2 encompasses the frequency of natural disasters and personal traumatic events. A certain number of participants experienced the same type of life event multiple times across the lifespan. With respect to natural disasters, 1819 (58.39%) experienced typhoon more than once. This proportion is lower in earthquake (11.40%) and tornado (2.73%). Death of a loved one is the most frequently experienced personal traumatic event and about half of participants (51.04%) experienced death of a loved one at least twice, followed by abortion (6.72%), miscarriage (3.27%), being robbed (2.27%), physical assault (1.60%), residential fire (0.46%), sexual assault (0.35%), cancer (0.32%), divorce (0.25%), false accusation of a crime (0.19%), homelessness (0.19%), and being imprisoned (0.09%).

A small proportion of participants considered natural disasters to be very serious: tornado (39.38%), typhoon (29.18%), and earthquake (16.30%). Regarding the personal traumatic events, 79.10% of participants considered being falsely accused as very serious, then 67.35% for being homeless, 65.22% for being imprisoned, 60.40% for experiencing the death of a loved one, 47.31% for being physical assaulted, 40.88% for having cancer, 40.66% for being robbed, 38.71% for experiencing sexual assault, 36.65% for divorce, 36.36% for experiencing a residential fire, 26.17% for miscarriage, and 19.72% for abortion. Among the historical events, 50.56% of the participants who experienced famine found the event to be very serious, followed by 48.69% for the Japanese invasion of China, 41.67% for the Cultural Revolution, 40.94% for the Vietnam war, and 40.90% for the Great Leap Forward (Table 3).

### 3.3. Correlation between Sociodemographic Factors and Life Events

Table 4 shows the correlation between demographic factors (age, gender, education, and income) and traumatic events (natural disasters, personal traumatic events, and historical events). The experience of earthquakes (r = −0.09, *p* < 0.001), typhoons (r = −0.06, *p* < 0.01), robberies (r = −0.08, *p* < 0.001), sexual assault (r = −0.05, *p* < 0.05), divorce (r = −0.10, *p* < 0.001), abortion (r = −0.11, *p* < 0.001), and Cultural Revolution (r = −0.10, *p* < 0.001) were more likely to be reported by younger participants. Other traumatic events, such as deaths of their loved ones (r = 0.07, *p* < 0.001), homelessness (r = 0.10, *p* < 0.001), false accusation of a crime (r = 0.08, *p* < 0.001), Japanese invasion of China (r = 0.61, *p* < 0.001), famine (r = 0.13, *p* < 0.001), and the Great Leap (r = 0.09, *p* < 0.001) were more likely to be reported by older participants. Males were more likely to experience typhoons (r = −0.05, *p* < 0.01), physical assault (r = −0.08, *p* < 0.001), being falsely accused (r = −0.09, *p* < 0.001), imprisonment (r = −0.06, *p* < 0.001), Japanese invasion of China (r = −0.05, *p* < 0.01), famine (r = −0.05, *p* < 0.01), the Great Leap (r = −0.08, *p* < 0.001), the Vietnam War (r = −0.06, *p* < 0.01), and the Cultural Revolution (r = −0.05, *p* < 0.05). Those who experienced sexual assault (r = 0.07, *p* < 0.001), miscarriage (r = 0.21, *p* < 0.001), abortion (r = 0.28, *p* < 0.001), and the death of a loved one (r = 0.10, *p* < 0.001) were more likely to be females.

The self-reported experiences of earthquakes (r = 0.06, *p* < 0.01), physical assault (r = 0.10, *p* < 0.001), sexual assault (r = 0.05, *p* < 0.01), divorce (r = 0.13, *p* < 0.001), abortion (r = 0.08, *p* < 0.001), death of loved ones (r = 0.15, *p* < 0.001), cancer (r = 0.14, *p* < 0.001), and false accusation of a crime (r = 0.08, *p* < 0.001), Great Leap Forward (r = 0.07, *p* < 0.001), and the Cultural Revolution (r = 0.08, *p* < 0.001) were correlated with older adults with higher education, while the experience of typhoons (r = −0.28, *p* < 0.001), tornadoes (r = −0.07, *p* < 0.001), the Japanese invasion of China (r = −0.04, *p* < 0.05), and the Vietnam war (r = −0.04, *p* < 0.05) were correlated with older adults with lower education. Exposure to typhoons (r = 0.04, *p* < 0.05) and divorce (r = 0.07, *p* < 0.001) was more likely to be reported by older adults with a higher income, while exposure to earthquakes (r = −0.08, *p* < 0.001), tornadoes (r = −0.04, *p* < 0.05), famine (r = −0.15, *p* < 0.001), the Great Leap (r = −0.16, *p* < 0.001), and the Cultural Revolution (r = −0.13, *p* < 0.001) was more likely to be reported by those with a lower income.

## 4. Discussion

Our study was among the first to examine life events among older Chinese Americans. We evaluated 20 life events, tapping into natural disasters, personal traumatic events, and historical events. The most prevalent, frequent, and severe life events experienced by the study population were the Cultural Revolution, typhoon, and falsely accused, respectively. Age, gender, education, and income were significantly correlated with lifetime experience of traumatic events.

With regard to personal traumatic events, the most prevalent event was death of a loved one, with 69.77% of older Chinese Americans with exposure to it. Death of a loved one has also been identified as the most prevalent life event in another study with a major focus on white older adults in the United States [30]. The prevalence rate of certain personal traumatic events might be affected by culture. For example, the prevalence rate of divorce among older Chinese Americans was 5.17%, which is higher than the rate in Chinese older adults in Singapore (1.5%) [16] and lower than white older adults in the United States (20%) [5]. In Asian culture, divorce is associated with sigma, which may lead to the low divorce rate in the Singapore study. The family norms of older Chinese Americans are influenced by both heritage culture and receiving culture, which may partially explain why the divorce rate in older Chinese Americans is higher than Chinese older adults in Singapore, but lower than natives in the United States. Meanwhile, the exposure to some personal traumatic events is related to the boarder environment. The prevalence of physical assault among Chinese older adults in Chicago (5.36%) and predominately white older adults in North Carolina (5.37%) [30] and the contiguous United States (3.8%) (Krause, Shaw et al. 2004) are all higher than the prevalence in Singapore (1.6%) (Lim, Lim et al. 2015). This difference is likely due to the crime rates in these societies.

The most prevalent natural disaster event experienced is typhoons (64.45%) in our study. These findings may be partially explained by where the participants migrated from. The majority of the participants came from the East Coast of China where typhoons are frequent due to its proximity to the Pacific Ocean. Thus, our participants had a high prevalence and a more frequent exposure to this natural disaster. One study in North Carolina showed that only 7.08% of white older adults experienced natural disasters [30]. In our study, 39.79% had experienced an earthquake, 64.45% experienced at least one typhoon, and 7.25% experienced a tornado. The exposure to natural disasters is often reliant on geological surroundings.

The historical events evaluated in our study took place in China, and thus their exposure to historical events depends on when they born and when they migrated to the United States. The earlier they born and the later they migrated, the greater number of historical events they were able to experience.

In addition to prevalence and frequency of lifetime traumatic events, this study further evaluated the subjective severity of each traumatic event amongst this study population. Existing research on lifetime traumatic events has assessed relationships with PTSD and depressive symptoms [31]. However, individuals were exposed to multiple traumatic events across the lifespan. Global measures for response to traumatic events (e.g., PTSD and depressive symptoms) leave us with incomplete knowledge, as they do not assess individual impact of each type of traumatic event [32]. There are heterogeneities in individual characteristics and supportive environment among older adults who have experienced the same type of traumatic event. The varying levels of severity of a life event reported by participants might be affected by their personality, cultural norms, and support systems [33,34,35].

Age, gender roles, and social economic status were related to exposure to traumatic events over the life course. Older age does not necessarily mean greater exposure to natural disasters, personal traumatic events, and historical events. Instead, different age cohort matters. One study among English-speaking older adults in the United States also found that the oldest old do not experience significantly more lifetime traumas than the young-old [5]. Older women in the study population were more prone to experiencing family and marriage related life events, such as sexual assault, miscarriages, and abortions, while older men were more likely to be exposed to traumatic events occurring outside of the family environment, such as being falsely accused of a crime and imprisonment. Similar patterns have been observed in a prior study, which reported that men were more likely than women to report having been physically assaulted by a stranger, while women were more likely to report intimate partner violence [32]. Older women are also more likely to experience deaths of a loved one than men, which is consistent with one study in Canada [1]. It is likely due to women having average longer life expectancies. Evidence is mixed regarding the relationship between social economic status and exposures to traumatic events. A literature review summarized that low social economic status was associated with increased exposures to traumatic events [17]. An empirical study reported that education was inversely associated with causing/witnessing bodily harm, experiencing interpersonal violence, having accidents/injuries, and the unexpected death of a loved one, but positively associated with exposure to collective violence, being mugged, automobile accidents, and sexual assault [18]. Interestingly, our results showed that older adults with higher levels of education had higher exposure to personal traumatic events, while income was not significantly related to most personal traumatic events. Education might influence an individual’s belief and behaviors and in turn affect one’s exposure to traumatic events.

The findings should be interpreted with caution. First, the experience of lifetime traumatic events was self-reported by participants. This may introduce recall bias, especially given older age. There is debate on the accuracy of retrospective reporting. Some maintain that these reports are flawed [36], while others demonstrate satisfactory validity and reliability in retrospective measures [37,38]. Second, this study focused on Chinese older adults living in the greater Chicago area. The findings might not be generalized to Chinese older adults living in different areas or to other age cohorts. The findings can also not be generalized to other ethnic groups. Third, this study did not assess the duration of each traumatic life event and event centrality [39], which may also influence trauma responses. Fourth, the severity of traumatic events was reported at the time of interview, not at the time when the traumatic event took place. On the one hand, the severity of traumatic event reported by participants during an interview might be different from their responses at the time of encountering the traumatic event. On the other hand, the subjective interpretation for severity could be the result of comparing cumulative traumatic events across the lifespan.

Despite these limitations, this study has important theoretical and practical implications. This study highlights the study population as a distinction from existing literature focusing on lifetime trauma in predominately white older adults. To the best of our knowledge, this study is among the first to examine traumatic events throughout the lifespan of older Chinese immigrants. An immigrant will likely experience traumatic events in both their homeland and in the country which they immigrated to. This study also encompasses 20 traumatic events, tapping into natural disasters, personal traumatic events, and historical events. There are heterogeneities among older adults who experienced lifetime traumatic events, manifesting by varying levels of prevalence, frequency, and subjective severity. Our findings could help identify which vulnerable groups have high risks of exposure to traumatic events. Future research on lifetime traumatic events could incorporate different ethnic groups to test whether there are racial and ethnic disparities in the exposure to traumatic events.

## 5. Conclusions

This research profiled the lifetime exposure to natural disasters, personal traumatic events, and historical events among older Chinese Americans. Older immigrants experienced traumatic events before and after migration. Many of them experienced more than one type of traumatic events and have had repeated exposed to certain traumatic events over their life course. Exposure to lifetime traumatic events differs by age cohorts, gender, and social economic status. A descriptive epidemiological study of lifetime trauma across racial and ethnic groups could help understand trauma and resilience in minority aging populations. This study could help health care professionals and social service agencies to identify which groups have higher trauma risks. The findings could inform trauma interventions to develop culturally relevant intervention strategies. Although the results may differ, this study can provide as a foundation to future research on trauma experienced by this cohort of Chinese immigrants in other regions of the United States. Future studies could examine the influence of cumulative exposure to traumatic events across the lifespan on the health outcomes of older immigrants.

## Figures and Tables

**Table 1:** Prevalence of life events (*N* = 3125).

Life Events	*N* (%)
**Natural Disasters**	
Earthquake	1240 (39.79%)
Typhoon	2009 (64.45%)
Tornado	226 (7.25%)
**Personal Life Events**	
Fire (residential)	165 (5.29%)
Physical Assault	167 (5.36%)
Robbery	391 (12.54%)
Sexual Assault	31 (0.99%)
Divorce	161 (5.17%)
Miscarriage	214 (11.26%)
Abortion	360 (18.92%)
Death of a loved one	2176 (69.77%)
Cancer	159 (5.10%)
Homeless	49 (1.57%)
Imprisonment	23 (0.74%)
Falsely Accused	67 (2.15%)
**Historical Life Events**	
Japanese invasion of China	846 (27.15%)
Famine	1871 (60.03%)
Great Leap Forward	1954 (62.73%)
Vietnam War	149 (4.78%)
Cultural Revolution	2282 (73.26%)

**Table 2:** Frequency of life events (*N* = 3125).

Life Event	*N* (%)	Life Event	*N* (%)
**Natural Disasters**		**Personal Life Events (Continued)**	
Earthquake		Miscarriage	
0	1876 (60.19%)	0	1687 (88.74%)
1	886 (28.42%)	1	152 (8.00%)
2+	355 (11.40%)	2+	62 (3.27%)
Typhoon		Abortion	
0	1108 (35.56%)	0	1543 (81.08%)
1	189 (6.07%)	1	232 (12.19%)
2+	1819 (58.39%)	2+	128 (6.72%)
Tornado		Death of a loved one	
0	2892 (92.75%)	0	943 (30.25%)
1	141 (4.52%)	1	583 (18.70%)
2+	85 (2.73%)	2+	1591 (51.04%)
**Personal Life Events**		Cancer	
Fire		0	2960 (94.90%)
0	2953 (94.71%)	1	149 (4.78%)
1	151 (4.84%)	2+	10 (0.32%)
2+	44 (0.46%)	Homeless	
Physical Assault		0	3069 (98.43%)
0	2950 (94.64%)	1	43 (1.38%)
1	117 (3.75%)	2+	6 (0.19%)
2+	50 (1.60%)	Imprisonment	
Robbery		0	3095 (99.26%)
0	2727 (87.46%)	1	20 (0.64%)
1	320 (10.26%)	2+	3 (0.09%)
2+	71 (2.27%)	Falsely Accused	
Sexual Assault		0	3051 (97.85%)
0	3086 (99.01%)	1	61 (1.96%)
1	20 (0.64%)	2+	6 (0.19%)
2+	11 (0.35%)		
Divorce			
0	2956 (94.83%)		
1	153 (4.91%)		
2–3	8 (0.25%)		

**Table 3:** Severity of life events (*N* = 3125).

Life Event	Severity of Life Event		
	Not Serious	Somewhat Serious	Very Serious
**Natural Disasters**			
Earthquake	828 (66.83%)	209 (16.87%)	202 (16.30%)
Typhoon	950 (47.31%)	472 (23.51%)	586 (29.18%)
Tornado	107 (47.35%)	30 (13.27%)	89 (39.38%)
**Personal Life Events**			
Fire (residential)	73 (44.24%)	32 (19.39%)	60 (36.36%)
Physical Assault	45 (26.95%)	43 (25.75%)	79 (47.31%)
Robbery	115 (29.41%)	117 (29.92%)	159 (40.66%)
Sexual Assault	9 (29.03%)	10 (32.26%)	12 (38.71%)
Divorce	64 (39.75%)	38 (23.60%)	59 (36.65%)
Miscarriage	120 (56.07%)	38 (17.76%)	56 (26.17%)
Abortion	217 (60.28%)	72 (20.00%)	71 (19.72%)
Death of a loved one	436 (20.06%)	425 (19.55%)	1313 (60.40%)
Cancer	52 (32.70%)	42 (26.42%)	65 (40.88%)
Homeless	11 (22.45%)	5 (10.20%)	33 (67.35%)
Imprisonment	6 (26.09%)	2 (8.70%)	15 (65.22%)
Falsely Accused	7 (10.45%)	7 (10.45%)	53 (79.10%)
**Historical Life Events**			
Japanese invasion of China	329 (39.07%)	103 (12.23%)	410 (48.69%)
Famine	499 (26.70%)	425 (22.74%)	945 (50.56%)
Great Leap Forward	758 (38.85%)	395 (20.25%)	798 (40.90%)
Vietnam War	72 (48.32%)	16 (10.74%)	61 (40.94%)
Cultural Revolution	906 (39.74%)	424 (18.60%)	950 (41.67%)

Note. *N* (%) was reported.

**Table 4:** Correlation matrix between study variables.

	Age	Female	Edu	Income	Earthquake	Typhoon	Tornado	Fire	PA	Rob	SA	Divorce	Miscarriage	Abortion	Death	Cancer	Homeless	Imprisonment	FA	JIC	Famine	GLF	Vietnam War	CR
Age	1.00																							
Female	−0.05 ^#^	1.00																						
Education	−0.05 ^#^	−0.17 *	1.00																					
Income	−0.08 *	0.01	0.04 ^+^	1.00																				
Earthquake	−0.09 *	−0.02	0.06 ^#^	−0.08 *	1.00																			
Typhoon	−0.06 ^#^	−0.05 ^#^	−0.28 *	0.04 ^+^	0.17 *	1.00																		
Tornado	0.03	0.02	−0.07 *	−0.04 ^+^	0.16 *	0.11 *	1.00																	
Fire	−0.02	−0.01	0.01	−0.01	0.08 *	0.02	0.04 ^+^	1.00																
PA	−0.03	−0.08 *	0.10 *	0.01	0.09 *	0.00	0.01	0.12 *	1.00															
Rob	−0.08 *	−0.01	0.03	0.02	0.11 *	0.02	0.07 *	0.02	0.16 *	1.00														
SA	−0.05 ^+^	0.07 *	0.05 ^#^	0.02	0.04 ^+^	0.00	0.03	0.08 *	0.08 *	0.08 *	1.00													
Divorce	−0.10 *	−0.03	0.13 *	0.07 *	0.03	−0.06 ^#^	0.01	0.04 ^+^	0.09 *	0.05 ^#^	0.08 *	1.00												
Miscarriage	−0.03	0.21 *	0.02	0.01	0.07 *	−0.04 ^+^	0.06 *	0.05 ^+^	0.03	0.07 *	0.05 ^#^	0.01	1.00											
Abortion	−0.11 *	0.28 *	0.08 *	−0.03	0.10 *	−0.03	0.04 ^+^	0.07 *	0.03	0.06 ^#^	0.05 ^#^	0.03	0.04 ^+^	1.00										
Death	0.07 *	0.10 *	0.15 *	−0.01	0.20 *	−0.10 *	0.10 *	0.08 *	0.10 *	0.09 *	0.05 ^+^	0.03	0.08 *	0.14 *	1.00									
Cancer	0.01	0.01	0.14 *	−0.02	0.02	−0.08 *	0.01	0.03	0.02	0.03	0.04 ^+^	0.09 *	0.06 ^#^	0.04	0.07 *	1.00								
Homeless	0.10 *	−0.02	−0.02	−0.03	0.00	0.03	0.04	0.02	0.07 *	0.02	0.04 ^+^	−0.01	−0.02	−0.01	0.05 ^#^	0.01	1.00							
Imprisonment	0.03	−0.06 *	0.02	−0.01	0.01	−0.02	0.01	0.00	0.08 *	0.04	0.03	0.00	−0.01	−0.01	0.03	0.03	0.08 *	1.00						
FA	0.08 *	−0.09 *	0.08 *	−0.02	0.02	−0.05 ^+^	0.00	0.03	0.16 *	0.02	0.01	0.05 ^#^	0.01	0.02	0.05 ^#^	0.04 ^+^	0.13 *	0.22 *	1.00					
JIC	0.61 *	−0.05 ^#^	−0.04 ^#^	−0.03	0.02	0.04 ^+^	0.09 *	0.02	−0.01	0.02	−0.01	−0.09 *	−0.03	−0.07 *	0.12 *	0.03	0.11 *	0.02	0.08 *	1.00				
Famine	0.13 *	−0.05 ^#^	0.00	−0.15 *	0.18 *	0.06 *	0.10 *	0.02	0.03	0.03	−0.00	−0.04 ^+^	0.05 ^#^	0.07 *	0.13 *	0.01	0.05 ^#^	0.02	0.06 ^#^	0.23 *	1.00			
GLF	0.09 *	−0.08 *	0.07 *	−0.16 *	0.19 *	0.04 ^+^	0.07 *	0.02	0.03	−0.00	−0.00	−0.04 ^+^	0.02	0.08 *	0.16 *	0.01	0.02	0.01	0.07 *	0.16 *	0.63 *	1.00		
Vietnam War	0.01	−0.06 ^#^	−0.04 ^+^	−0.00	0.09 *	0.00	0.11 *	0.04 ^+^	0.05 ^#^	0.09 *	0.01	0.02	0.04 ^+^	0.02	0.03	−0.00	0.03	−0.00	0.01	0.05 ^#^	0.04 ^+^	0.01	1.00	
CR	−0.10 *	−0.05 ^+^	0.08 *	−0.13 *	0.21 *	0.07 *	0.05 ^+^	0.01	0.03	−0.01	−0.02	−0.03	0.02	0.10 *	0.08 *	−0.02	0.00	0.03	0.07 *	0.05 ^#^	0.47 *	0.63 *	−0.01	1.00

Note. PA= Physical assault, SA= Sexual assault, JIC = Japanese invasion of China, FA = Falsely accused, GLF = Great Leap Forward, CR = Cultural Revolution. ^+^ *p* < 0.05, ^#^ *p* < 0.01, * *p* < 0.001.

## Data Availability

The data presented in this study are available on request from the corresponding author.
